# Optimizing Cellular Metabolism Through Mass Balance Analysis to Improve Skin Wound Healing

**DOI:** 10.3390/biology14060722

**Published:** 2025-06-18

**Authors:** Luis Ramirez Agudelo, Gabriel Yarmush, Suneel Kumar, Francois Berthiaume

**Affiliations:** Department of Biomedical Engineering, Rutgers, The State University of New Jersey, Piscataway, NJ 08854, USA; lfr30@connect.rutgers.edu (L.R.A.); gabriel.yarmush@gmail.com (G.Y.); sk1350@soe.rutgers.edu (S.K.)

**Keywords:** wound healing, metabolic flux analysis, keratinocytes, cell proliferation, ATP synthesis

## Abstract

Accelerating healing is a clinical goal in both acute and chronic non-healing skin wounds. We leveraged the public Recon database, which seeks to aggregate all of the metabolic pathways in the human body, to uncover whether increasing the supply of specific metabolites can bolster cellular metabolism and, in turn, enhance wound healing. The database was reduced to a set of 357 reactions and 339 metabolites that were better suited for human cells in culture. Monte Carlo simulations were performed to identify the impact of 25 different inputs on the metabolic fluxes within the cellular biochemical network. Biomass and ATP production were used as surrogate markers for cell proliferation and cell migration (an energy-intensive process), respectively, both of which are critical to wound healing. The subset of simulations yielding the highest ATP production or biomass production were those where glycine and/or glutamine uptake was increased. Maximizing ATP and biomass also generally increased oxygen uptake. Due to its low availability in chronic wounds, another set of simulations was carried out in which oxygen uptake was held constant to mimic the effect of a limited oxygen supply. However, even with this constraint, glycine and glutamine remained the most promising interventions. The predictions were tested in vitro using immortalized human keratinocytes. Amino acid uptake was tentatively increased by supplementing the base culture media with additional glycine and/or glutamine, with valine supplementation with a similar nitrogen load as a control. Glycine supplementation significantly increased cellular proliferation above the base media and accelerated wound closure rate in wound scratch assay. However, glutamine and valine supplementation did not improve these parameters above base media, and glutamine even suppressed the benefit of glycine in cultures supplemented with both amino acids. In conclusion, glycine supplementation enhances cellular processes that are associated with wound healing.

## Simple Summary

Chronic wound healing remains a significant clinical challenge despite years of intense research. This study leverages insights from known cellular biochemistry using computational modeling to identify potential metabolic supplements that could improve chronic skin wounds. In particular, the Recon database was analyzed computationally to identify glycine and glutamine as the most likely metabolites to enhance ATP and biomass synthesis during both unrestrained and restrained oxygen uptake, which more closely mimics hypoxia in chronic skin wounds. This finding was subsequently tested in vitro on cultured immortalized keratinocytes, and revealed that glycine significantly increased cellular proliferation and accelerated wound closure. Glutamine and valine, used to control for nitrogen load increase, showed no improvement in these parameters.

## 1. Introduction

Factors known to delay wound healing include impaired peripheral circulation, advanced diabetes, old age, infection, and poor nutrition [1,2,3]. Several strategies are currently used in the clinic to aid wound healing, such as specialized wound dressings, negative pressure wound therapy, topical growth factors, and hyperbaric oxygen, to name a few [4,5,6,7]. These approaches mainly focus on providing biochemical signals via extracellular matrix and growth factor molecules, and do not address the increased metabolic demands of the cells in the wound environment [8]. Hyperbaric oxygen may increase oxygen delivery locally, although the literature suggests that it may act primarily by stimulating the local generation of growth factors [9,10]. Nutritional status is generally viewed as critical to wound healing, and there have been a few studies providing nutrients to the wounds topically. In particular, glycine and proline, two major components of collagen, as well as the tripeptide arginine–glycine–aspartic acid (RGD), have been used in an attempt to increase cellular production of extracellular matrix and in the wound. However, wound healing is also driven by cell migration and proliferation, two highly energy-intensive processes, thus requiring an increased supply of ATP [11].

Much research has gone into optimizing the metabolism of a variety of cells. For example, in yeast, researchers have attempted to modify the metabolic pathways of the cell to permit the production of biofuels from lignocellulosic biomass [12]. Metabolic flux analysis, which accounts for stoichiometric relationships among metabolites in the totality of a cell’s metabolic network, can be used to explore the impact of changing uptake rates of individual or combinations of extracellular metabolites on intracellular processes. Metabolic flux analysis was used to determine how tumor cells with mutations in key mitochondrial enzymes can support biosynthetic activities [13]. Faubert et al. also used metabolic flux analysis in cancer patients to show that lactate metabolism plays a key role in feeding the tricarboxylic acid cycle and thereby affects tumor cell metabolism in vivo [14].

In this study, we explored the impact of changing the uptake of 25 different extracellular metabolites on ATP production and biomass production, using a stoichiometric model of the major reactions that handle carbon and nitrogen in the cell. ATP and biomass output parameters were chosen as proxies in the model for cell migration and cell proliferation, respectively, two major processes involved in wound healing. We used the publicly available Recon database as a starting point, which we reduced to a set of 357 reactions that could be used to explore metabolite inputs that increase fluxes of ATP and biomass production using a metabolic flux analysis framework [15,16]. We then experimentally verified the effect of increasing the availability of two amino acids (glycine and glutamine), which were identified as potentially beneficial, on human keratinocyte proliferative and healing responses.

## 2. Materials and Methods

### 2.1. Materials and Equipment

Phosphate-buffered saline (PBS, Gibco, Waltham, MA, USA), HaCaT cells (P47–54, Life Technologies, Carlsbad, CA, USA); Dulbecco’s modified Eagle medium (DMEM; Thermo Fisher Scientific, Waltham, MA, USA); 10% *v*/*v* fetal bovine serum (FBS; Atlanta Biologicals Flowery Branch, GA, USA); 1% Pen/Strep (Life Technologies, Carlsbad, CA, USA); valine (Sigma-Aldrich, V0500, Saint Louis, MO, USA); glutamine (Sigma-Aldrich, G8898); glycine (Sigma-Aldrich, G8540); AlamarBlue (ThermoFisher Scientific, USA); inverted microscope (Olympus CKX41, Infinity 2 Lumenera camera, Ottawa, ON, Canada); ImageJ (NIH version 1.53, 2021); GraphPad (10.4.2;633).

### 2.2. Wound Healing Metabolic Network Construction

To build a metabolic network representative of the cellular metabolism in wounds, the Recon 2 database, which seeks to recompile all of the intracellular metabolic reactions in the human body (consisting of 7785 reactions involving 5324 different metabolites) was used as a starting point [16]. The database was reduced to a smaller set of reactions more appropriate to human cells in culture, consisting of 357 reactions involving 339 metabolites, using a systematic approach described by Quek et al. [15]. In this process, the main cellular metabolic pathways were emphasized to make the system of reactions solvable upon the measurement of a limited (25) extracellular flux [15]. The reduction process involves four steps: (1) reserved functions, (2) futile adenosine triphosphate (ATP) metabolism, (3) redundant reactions, and (4) loopless reactions. In the first step, certain types of reactions were constrained. This was achieved by manually identifying all of the reactions involved in key processes such as ATP and nicotinamide adenine dinucleotide phosphate {NAD(P)H} production, the transport of compounds across the cell membrane, and the fixation of nitrogen. Each category of reactions was constrained such that only the reaction identified as having the greatest flux with flux variability analysis was included. Moreover, reactions that are not relevant to cells in culture, such as transport of compounds that do not present in cell culture media, had their flux values set to 0.

In the second step of the reduction process, reactions that consume ATP without a clear metabolic purpose were identified and grouped into a category termed “ATP maintenance.” Some of the reactions that cyclically consume ATP were set to zero after performing flux variability analysis to assess their importance while trying to maximize biomass. In the third step, reactions that are redundant as a result of intracellular compartmentalization, or because they can substitute different cofactors (e.g., NADH or NADPH), were identified. The set of reactions was decomposed into a minimum number of sub-models according to a flux constraint. Within each sub-model, only one reaction was retained. In the final step, thermodynamically infeasible reactions were identified using flux variability analysis. These reactions may have been infeasible at the outset, or they may have become infeasible as a result of the reduction steps. This process of reduction was entirely carried out using MATLAB (Mathworks R_2017).

### 2.3. Metabolic Flux Analysis

The reduced reaction database is a list of chemical reactions describing the stoichiometric relationship between reactants and products. This list is expressed as a matrix and was used as the starting point for metabolic flux analysis. The key assumptions of this metabolic flux analysis model are (1) the existence of a steady state and (2) the existence of a single intracellular compartment where all reactions take place. To carry out the subsequent task, the flux through a subset of 25 key reactions that mostly involve extracellular metabolites exchanging between the extracellular medium and the inside of the cell was arbitrarily specified as inputs to the model. More specifically, the 25 reactions include the exchange reactions for every amino acid except cysteine, the exchange reactions for glucose, lactate, and ammonia, and oxygen uptake (Table S1). Although biomass is not exchanged across the cell membrane, for this analysis, it was assumed to accumulate at a constant rate in a compartment separate from the cellular reaction network. The specified values used here are those measured previously by Quek et al. [15] and are provided in Table S2. The number of specified inputs is sufficient to yield a unique solution when solving the stoichiometric matrix, thus providing the flux through all of the unknown reactions in the intracellular metabolic network that satisfy the mass balance constraints of the stoichiometric matrix.

Mathematically, we solved the equation vector for a subset of v in the equation *S∙v =* 0, where S is the stoichiometric matrix and v is the vector of metabolic fluxes [17]. The equation in the previous sentence also arises because of the assumption of mass balance at a pseudo-steady state. One can then use the additional assumption that the m subset of the fluxes are the specified extracellular fluxes. From this assumption, the stoichiometric matrix can be divided into $S_{specified}$ and $S_{unknown}$, such that $S_{specified}$∙$v_{specified}$ + $S_{unknown}$∙$v_{unknown}$ = 0 [17]. One can then rewrite this equation as *v_unknown_ = (S_unknown_∙S_known_’)^−^*^1^*∙S_specified_∙V_specified_.* From this last equation, one can explicitly solve for $v_{unknown}$, or the unknown fluxes, with a set of known or specified variables.

### 2.4. Effect of Varying Inputs on Flux Model

To identify the extracellular metabolites that have the most impact on biomass production and ATP synthesis, each input of extracellular metabolite was expressed as an average plus or minus a standard deviation of ±10%. The metabolic flux analysis framework was used to solve the 357 reactions using, as a starting point, the measured extracellular metabolite rates reported by Quek et al. [15], except biomass, lactate, and ammonia. For each simulation, the measured metabolite uptake rates had their values specified randomly in a normal distribution with a mean value equal to that previously reported by Quek et al., and a standard deviation of 10% of the mean. For each simulation, the vector of unknown fluxes was solved. All the calculations involved in this step were carried out on MATLAB (Mathworks R_2017).

Since we did not initially know how many simulations would be needed to obtain a unique solution, we first carried out batches of 10 simulations with randomly selected flux numbers each time and compared the results among the 10 simulations. This was repeated using batches of 100, 1000, and 10,000 simulations. The criterion used for convergence was that the ratio of standard deviation to the mean for biomass production should be less than 5%. This method was used to determine how many simulations to use for the remainder of the computational work.

#### 2.4.1. Effect of Varying All Extracellular Metabolites Simultaneously on ATP and Biomass Production

The results of the convergence test were then used to perform a sensitivity analysis of the model. Then, the simulations with the highest ATP were explored as a marker for increased migratory ability in wound healing, and those with the highest biomass were used as a marker for increased proliferation. Based on the results of the convergence tests, 1098 simulations were run in which all of the extracellular metabolites (except for biomass, ammonia, and lactate) were varied around a mean equal to that reported by Quek et al., ±10% standard deviation. The code for these calculations was written in MATLAB. For the set of 1098 simulations, the average of each reaction flux and standard deviation (expressed as a percentage of the average flux value) over the 1098 simulations were calculated.

Then, simulations associated with the largest ATP production were further explored. Total ATP production was calculated for each simulation by adding fluxes for the two major reactions in the metabolic network that produce ATP, namely ATP synthase and pyruvate kinase. The 20 simulations that led to the largest ATP production were averaged. This was carried out in an attempt to select the simulations with the highest amount of ATP production. Then, the percentage difference in this “top 20” average relative to the average of all simulations was calculated as (flux through average of the 20 simulations with the highest ATP production-average for that reaction over a total of 1098 simulations)/(average for that reaction over a total of 1098 simulations). The same procedure was repeated to find the subset of 20 simulations that maximized biomass production.

#### 2.4.2. Effect of Varying All Extracellular Metabolites While Keeping Oxygen Uptake Fixed on ATP and Biomass Production

Because oxygen delivery may be constrained in the actual wound due to the patient’s pathology, we also examined simulation results when oxygen uptake was fixed and thus not allowed to change. A hypoxic level was simulated as 10% of free oxygen uptake in the model, as this is a similar magnitude change as occurs between normoxic and hypoxic cell culture conditions [18,19]. A one-to-two magnitude change has also been reported for in vivo hypoxic conditions [18]. Otherwise, the same procedure as outlined above for finding the 20 simulations out of 1098 with the highest ATP production, and then the highest biomass was performed.

### 2.5. Metabolic Activity/Viability of Keratinocytes (HaCaT)

HaCaT cells (P47–54) were grown using DMEM supplemented with 10% (*v*/*v*) FBS and 1% Pen/Strep. A 96-well plate was seeded at a density of 5000 cells/well. For the first 24 h after plating the cells, they were grown under normal DMEM conditions. At 24 h, the media was replaced with amino acid-supplemented media, which was prepared as described next. The DMEM with FBS and Pen/Strep was further supplemented with either valine (0.8–16.8 mM), glutamine (4–20 mM), glycine (0.4–4.0 mM), or both glutamine and glycine (8 mM of glutamine and 1.8 mM of glycine). Forty-eight hours later, 10% AlamarBlue reagent was added to the first set of wells. The plate was incubated for one hour before measuring the fluorescence intensity in each well using a plate reader (535 nm excitation/595 nm emission). Another set of wells was analyzed similarly at 72 h. A standard curve of known cell numbers per well (created by plating known cell numbers 24 h earlier) versus fluorescence was used to convert raw data into cell numbers per well. The results were then divided by the average cell number for the 24 h control (non-supplemented DMEM condition) [20].

### 2.6. Cell Migration Scratch Assay

HaCaT cells were plated in a 24-well plate (250,000 cells/well) and cultured in DMEM supplemented with 10% FBS and 1% Penn/Strep. A line was drawn horizontally on the underside of the plate with a marker before plating the cells. The cells were grown to confluence (90–100%) for 48 h, and a 200-microliter pipette tip was used to create a single scratch through the monolayer, perpendicular to the previously drawn line. The cells were imaged immediately after scratching (time 0) and every 6 h thereafter using an inverted microscope [20].

Wound images were analyzed using ImageJ. The area of the wound at each time point was measured using ImageJ. Percentage closure was calculated by subtracting the percentage change in wound area at time 0 from 100%, or (100% − ((wound area at time point)/(wound area at time 0) × 100)).

## 3. Results

### 3.1. Mathematical Modeling Reduction Process

**Figure 1:**
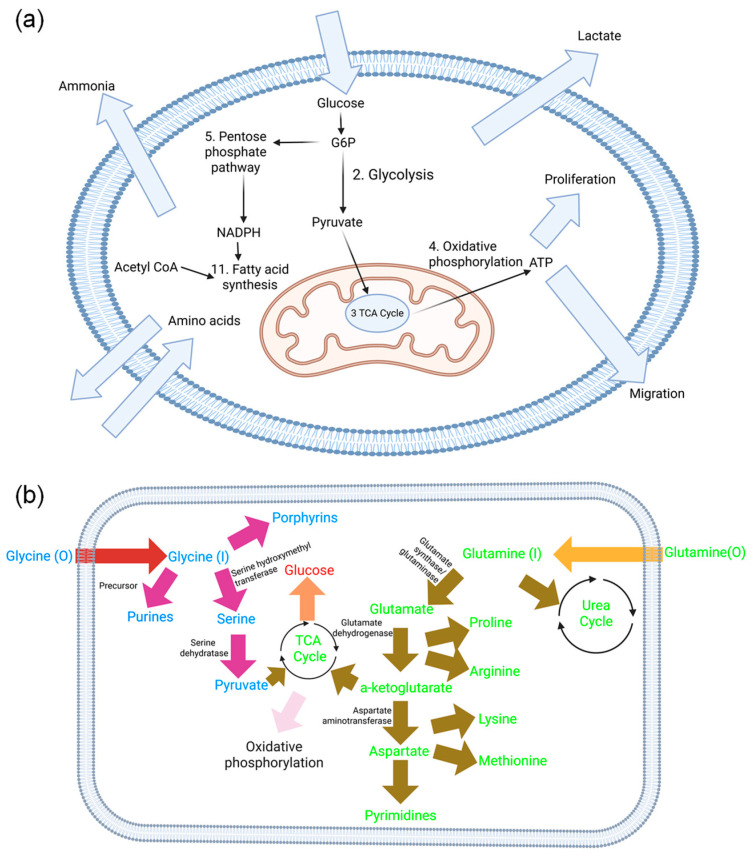
(**a**) Schematic of the main metabolic pathways in the cells that are included in the metabolic flux model. Key reactions are numbered according to their order in Table S3. (**b**) Shows the many metabolites and pathways that originate from glycine and glutamine within the cell. This could help explain the predicted effects of both amino acids, particularly glycine’s in vitro-measured effects. Different color arrows were used for glycine and glutamine products and for common pathways.

The cellular metabolic network was reduced according to the process described by Quek et al. [15], to yield a matrix of 357 reactions containing 339 metabolites. The summary of the reactions grouped by function and the full list of reactions are provided in Table S3. The flux through each reaction grouped by category using the fluxes from Quek et al. is shown in Figure S1 [15]. Below in Figure 1 is a summary of the main metabolic pathways accounted for in the model, which include glycolysis, the pentose phosphate pathway (PPP), the tricarboxylic acid (TCA) cycle, and oxidative phosphorylation (which includes the electron transport chain).

### 3.2. Mathematical Modeling Metabolic Flux Analysis

**Figure 2:**
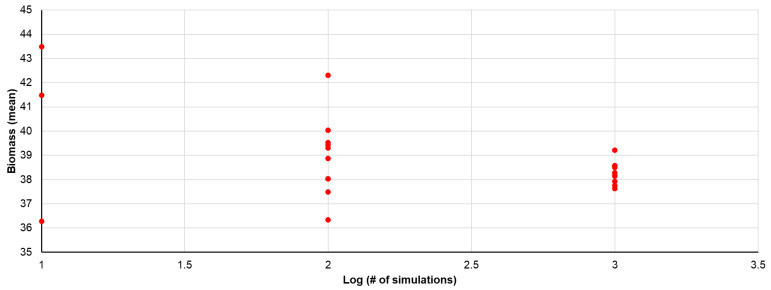
Mean biomass over 10 runs for a given number of simulations per run. The standard deviation was less than 5% of the average for the 10 runs of 1000 simulations. The data represent the mean biomass for a given number of simulations, varying only one extracellular metabolite at a time.

Metabolic flux analysis was employed to determine several properties. First, we assessed the agreement between our flux results and those reported in the original paper by Quek et al. (Table S4). There was consistency in 10 of the 12 reactions reported by Quek et al., with the exceptions being pyruvate dehydrogenase and glutamine transport into the cell [15]. For these two, the differences were minor, at only 2.9% relative to the range for glutamine transport and 8.8% relative to the range for pyruvate dehydrogenase. Next, we calculated the number of simulations necessary to yield consistent results. To do this, we determined the number of simulations using randomly selected input values within ±10% of the average for each that would produce a consistent rate of biomass production. Figure 2 illustrates the average biomass production rate predicted using random inputs for 10, 100, 1000, etc., independent simulations. The convergence of the results for increasing numbers of simulations is evident, with approximately 1000 simulations yielding an average biomass rate with less than 5% standard deviation among batches of 1000 simulations. For subsequent studies, we performed 1098 simulations at a time. An examination of the simulation data showed that ATP synthesis and biomass production were among the top 10 reactions with the highest average flux, as well as the highest relative standard deviation (Supplement C). This suggests that these fluxes would be highly sensitive to variations in input values, making them good candidates for optimization. The results of sensitivity analysis, which help pinpoint which reactions are the most impactful, are presented in Figures S2–S6.

### 3.3. Extracellular Metabolite Fluxes Maximize ATP Production

**Figure 3:**
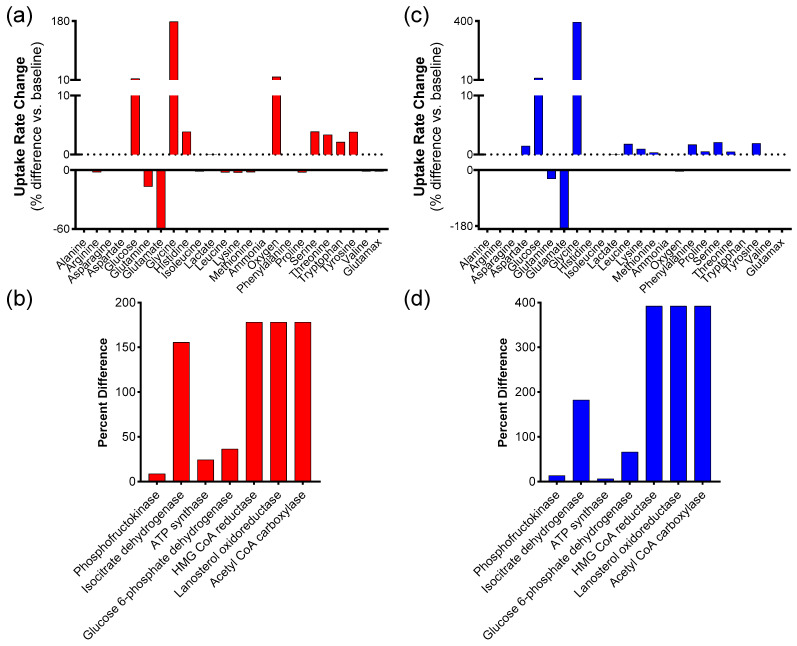
Simulations in which all extracellular metabolites, including oxygen uptake, were varied by ±10%. (**a**) Difference in transport reactions between the average of the 20 simulations with the highest ATP production versus the totality of 1098 simulations. Negative numbers indicate that less metabolite flowed into the cell, or that more metabolite flowed out of the cell. (**b**) Difference in flux relative to baseline through 7 representative reactions for the 20 simulations with the highest ATP production; these reactions correspond to different groups of reactions identified in Figure 1. Phosphofructokinase corresponds to glycolysis, isocitrate dehydrogenase corresponds to the TCA cycle, ATP synthase to oxidative phosphorylation, and glucose 6-phosphate dehydrogenase corresponds to PPP. HMG CoA reductase corresponds to cholesterol synthesis, lanosterol oxidoreductase corresponds to cholesterol synthesis, while acetyl CoA carboxylase corresponds to fatty acid synthesis. (**c**) Difference in transport reactions between the average of the 20 simulations with the highest biomass production vs. the totality of 1098 simulations. (**d**) Difference in flux relative to baseline through 7 representative reactions for the 20 simulations with the highest biomass production. These reactions correspond to the same groupings in Figure 1 as in (**b**).

Out of the 1098 simulations, the data for the subset of 20 simulations with the highest ATP production were averaged and compared to the baseline average for the entire data set. The average ATP production for the top 20 simulations was 2452 μmol/gDW/h, compared to the baseline of 1969 μmol/gDW/h for the complete 1098 simulation data set. The percentage difference in the input transport reactions between the averages of the top 20 and the entire simulation data set is shown in Figure 3a. Metabolite fluxes with significant changes include oxygen uptake and transport reactions for glycine, glutamate, glucose, and glutamine. Oxygen uptake and glycine uptake revealed substantial positive increases in flux into the cell, whereas glutamine and glutamate uptake showed declines (Table S5). Key changes in intracellular metabolic pathways were examined by comparing fluxes for representative reactions in glycolysis, the citric acid cycle, oxidative phosphorylation, the pentose phosphate pathway, cholesterol synthesis, steroid synthesis, and fatty acid synthesis (Figure 3b). The data indicate that all pathways increased in the high-ATP subset compared to the baseline, with the largest percentage observed in fatty acid, steroid, and cholesterol biosynthesis.

### 3.4. Extracellular Metabolite Fluxes-Maximize Biomass Production

**Figure 4:**
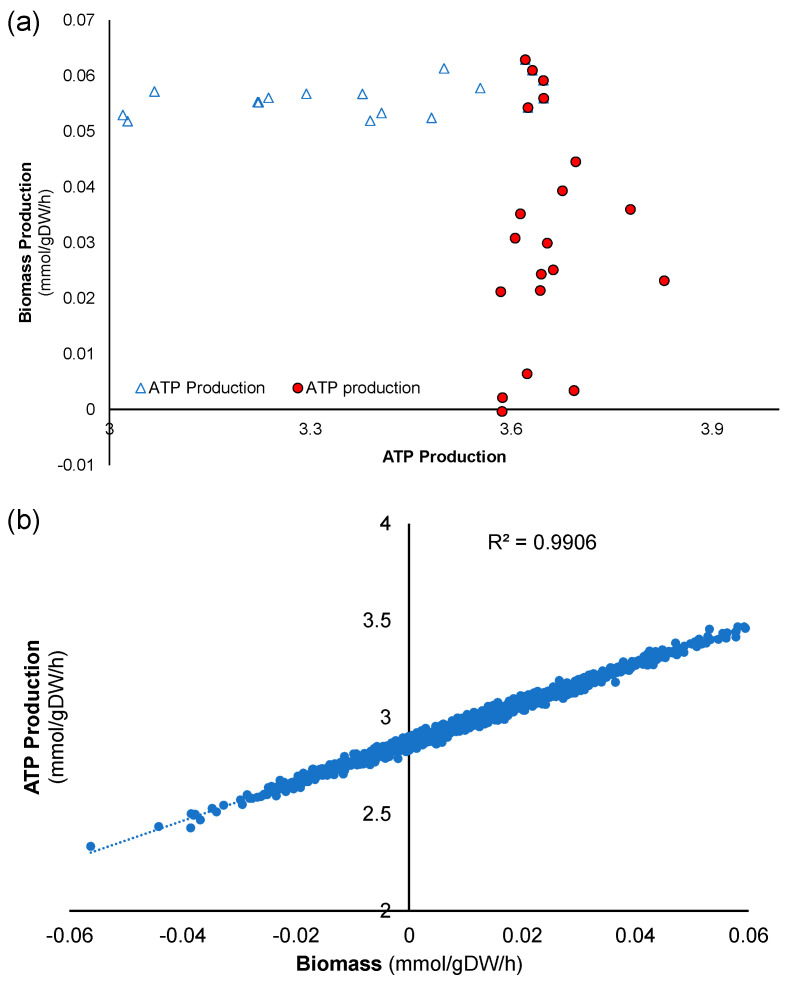
(**a**) Graph of ATP production versus biomass production for the 20 simulations that maximize biomass and the 20 simulations that maximize ATP production. (**b**) Correlation between ATP and biomass production for all simulations.

Out of the 1098 simulations, data for the subset of 20 simulations yielding the highest biomass production were averaged and compared with the overall average for the entire data set. The average biomass production for the “top 20” simulations was 56.25 μmol/gDW/h, compared to the baseline of 11.42 μmol/gDW/h for the entire 1098. The simulation dataset presents numerical values found in Table S6. Key changes in intracellular metabolic pathways were examined (Figure 3c), revealing results similar to those of maximizing ATP (Figure 3b). Figure 3d illustrates how the input fluxes for the 20 simulations with the highest biomass differ from the baseline average of all 1098 simulations. Glycine uptake increased, while glutamate uptake decreased, showing the largest relative changes (Table S7). Due to the similarities in input flux changes that maximize ATP and biomass, we investigated the extent of overlap between the two conditions; in other words, whether maximizing one automatically maximizes the other. For this purpose, each of the top 20 data points that maximize ATP and biomass (40 simulations total) is displayed in Figure 4a. The data points appear to converge in the top right corner, where approximately five simulations overlap, defining conditions that maximize both ATP and biomass simultaneously. Analyzing the entire data set of 1,098 simulations, one can observe that ATP production and biomass production are generally linearly correlated (Figure 4b), indicating that conditions that increase one also increase the other, and vice versa.

### 3.5. Maximizing ATP and Biomass Under Restricted Oxygen Uptake Conditions

Since many of the interventions seek to improve the healing of chronic and slow-healing wounds, which are largely hypoxic, we carried out simulations under the constraint that oxygen uptake was fixed and set to 10% of the baseline level used in prior simulations.

**Figure 5:**
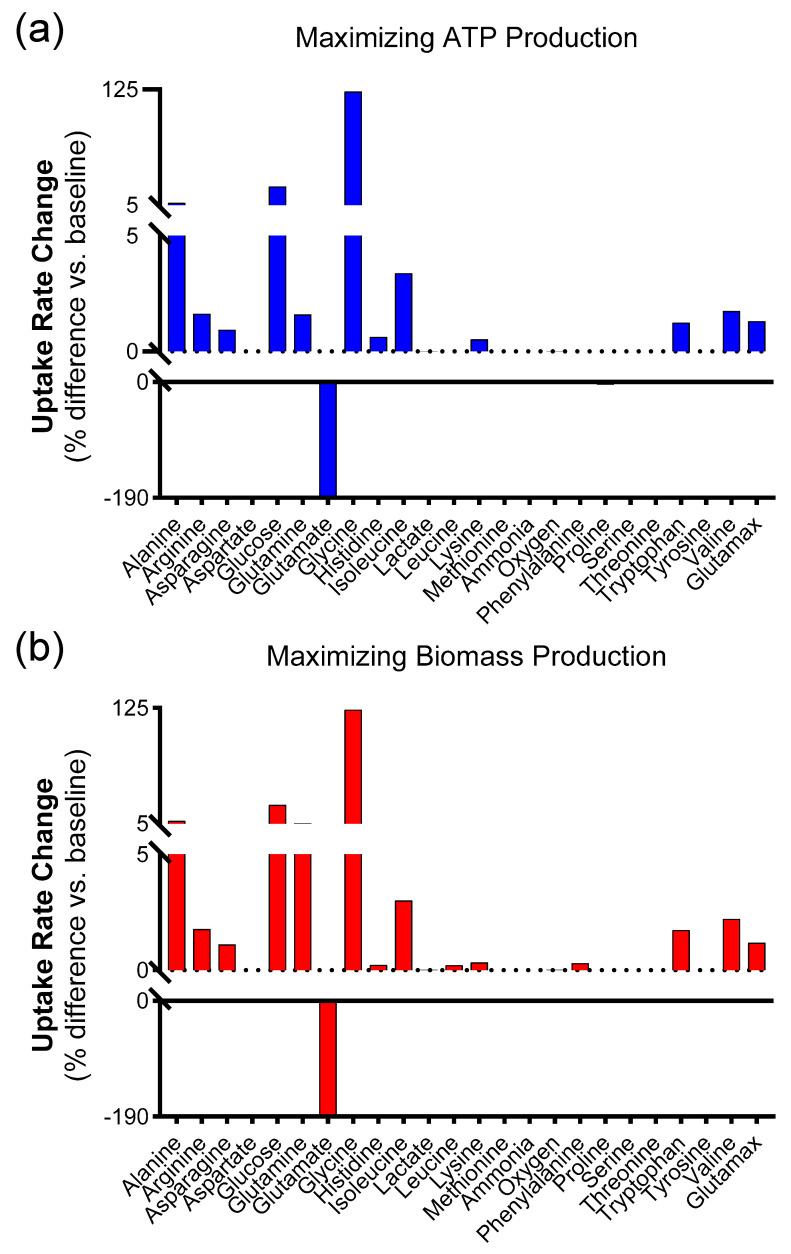
Simulations in which the oxygen uptake rate was set to 10% baseline and held constant. (**a**) Difference in transport reactions between the average of the 20 simulations with the highest ATP production versus the totality of 1098 simulations. (**b**) Difference in transport reactions between the average of the 20 simulations with the highest biomass production versus the totality of 1098 simulations.

Again, we identified the subset of 20 simulations that maximize ATP production, and then the subset that maximizes biomass production. The results, shown in Figure 5, indicate patterns similar to those observed in the absence of oxygen limitation (Figure 3), whereby these scenarios are associated with increased glycine and glucose uptake. Again, there was a significant decrease in the flux of glutamate into the cell. There was also a consistent increase in glycine uptake in Figure 5. Internal flux changes through representative pathways were also similar to those observed without oxygen restriction (Figures S7 and S8; Tables S8 and S9). These tables show that despite the large percentage difference associated with glycine, its total change is much smaller. This was also observed for the case in which oxygen was varied. Meanwhile, Tables S10 and S11 show the list of those reactions with the greatest percentage difference in flux compared to their flux for all 1098 reactions. This list shows a significant downregulation in branched-chain amino acid catabolism and particularly valine catabolism and transport into the mitochondria.

### 3.6. Effects of Amino Acids on Cells’ Metabolic Activity/Viability

The results show that glycine and glutamine are the best amino acids for further exploration (Figure 3 and Figure 5). In particular, the results for limited oxygen uptake showed consistently higher uptake for both amino acids. Some of the other increases, such as those for oxygen and glucose, are more obvious and are being attempted by other researchers [21,22,23,24]. The two best choices for a second amino acid to test were glutamine and alanine. Both amino acids showed slight decreases under normoxic conditions but were higher in limited oxygen conditions. Given the low-oxygen conditions of many skin wounds, such as chronic skin wounds, low-oxygen simulations were taken into greater consideration. They were both similar in their increases under low-oxygen conditions, so the choice of which one to test further was made based on their relative concentration in the base media used. In this regard, glutamine is present in higher concentrations. This means that a given increase in percentage will translate into a larger increase in the absolute amount. Given this consideration, glutamine was chosen for further exploration. Predictions from metabolic flux analysis were tested in a cell culture model consisting of keratinocytes, which are analogous to the cells of the skin epidermis. The first test was to see whether the amino acids glycine, glutamine, or a combination of the two would yield an increase in cell proliferation as suggested by the metabolic model. The level of glycine in the media was increased 10-fold while that of glutamine was increased 5-fold. Valine was increased 21-fold, and used as a control amino acid supplement, not expected to benefit ATP or biomass production by the keratinocytes. Meanwhile, the level of valine was increased to be equivalent, in terms of nitrogen load, to the increase in glutamine.

**Figure 6:**
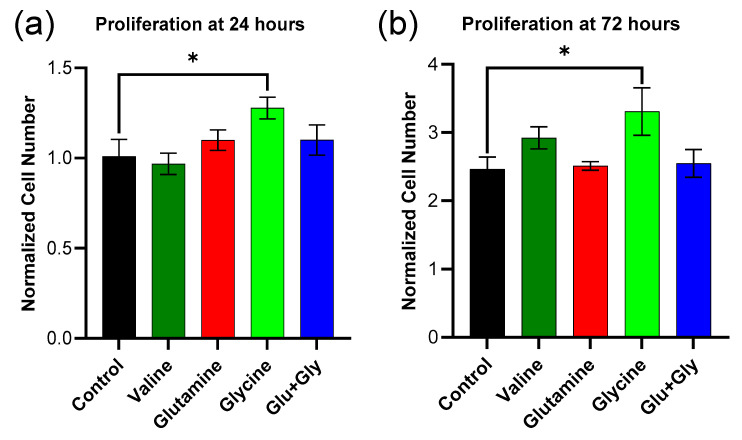
Effect of amino acid supplementation on HaCaT cell proliferation over the period of 72 h. Cell number normalized to initial after 24 (**a**) and 72 h (**b**) of incubation (*n* = 10) for all conditions. Data are represented as the mean ± standard error of the mean (SEM). Statistical analysis was performed using one-way ANOVA followed by Dunnett’s multiple comparisons test. * *p* < 0.05.

The results show significantly higher proliferation with glycine supplementation after both 24 and 72 h of culture (Figure 6a). Glutamine and valine showed a trend, but not statistically significant, towards an increase of 72 h. Interestingly, the combination of glutamine and glycine did not enhance proliferation, and the presence of glutamine seemed to suppress the benefit of glycine used alone.

**Figure 7:**
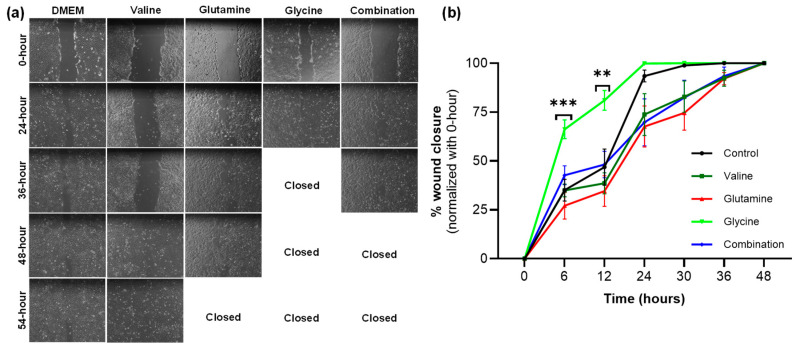
Effect of amino acids on scratch wound closure using HaCaT cells. (**a**) Representative microscopy images of scratch wounds are shown from different groups over 54 h. (**b**) The % wound closure was calculated to show the function of time. Data are presented as the mean ± SEM (n = 6–9). Statistical analysis was performed using two-way ANOVA. ** *p* < 0.01 and *** *p* < 0.001. Combination-glutamine and glycine together.

The second test was then to see if similar amino acid supplementations enhance the rate of wound closure using scratch wound assay on keratinocytes monolayers (Figure 7a). Wound closure kinetics were faster with glycine supplementation as compared to normal media control, and media supplemented with glutamine, valine, and the combination of glutamine and glycine (Figure 7b). More specifically, wound closure was significantly higher in the glycine group at both 6 h (*p* < 0.001) and 12 h (*p* < 0.01) and then wounds were completely closed by 24 h.

## 4. Discussion

This paper outlines the results of computationally probing a metabolic model of mammalian cells to ascertain which extracellular metabolites are most likely to improve skin wound healing. In particular, the model predicts that increasing glutamine and glycine uptake rates are associated with higher biomass and ATP production. These findings are significant because these markers—representing cell proliferation and migration, respectively—are intimately involved with the process of wound healing.

A choice was made to utilize the recon database, which is one of the most comprehensive databases available for metabolism, as well as a previously published paper that optimized the database for use with cells in culture. This allowed the researchers to utilize a limited set of previous measurements of metabolites in cell culture to computationally model flux through the entire metabolic model. Other models exist, such as 13-MFA, which provide alternate frameworks with added benefits and detractors. 13-MFA, for example, would allow for more precise measurements of flux through different pathways. In the end, however, making a more precise measurement through such a thorough metabolic network would require extensive work. An alternative could be limiting the model, but this would likely result in modeling that would not be as useful for finding a thorough solution to the problem. This can be obviated by utilizing simple measurements and using the metabolic model that was used in the paper.

Although the model suggests increasing glycine and glutamine fluxes, it does not specify how this can be achieved in reality. In this study, we attempted to enhance the uptake of these amino acids by increasing their concentration in the culture media, and we found that glycine increased both proliferation and wound closure rates in a scratch assay compared to both normal media and valine. It is worth noting that while it was predicted that glutamine would also lead to an improvement, the model predicted a much larger effect from glycine. The effects of glycine diminished at longer time points. Cell proliferation with glycine supplementation was not significantly different from DMEM at 72 h, presumably due to the consumption of glycine over that period. The lack of effect from glutamine might also be explained by its higher initial concentration in DMEM. Glutamine was initially present at a concentration of 4 mM, whereas glycine was present at 0.4 mM. Moreover, under normoxic conditions, the model showed a decrease in the transport of glutamine into the cell. The lack of effect under glutamine supplementation might also be explained by the fact that glutamine can be deaminated inside the cell to produce glutamate, which showed a marked decrease in the simulations that maximized ATP and biomass production.

It is worth noting that certain similarities between wound healing and cancer exist, particularly concerning increased cell proliferation and migration, which may manifest as metastatic behavior. Thus, these associations have also been implicated in the efficacy of cancer cell proliferation. In particular, increased glycine metabolism has been associated with higher cell proliferation and purine synthesis in cancer [25]. Likewise, glutamine has been found to support ATP production and protein synthesis in rapidly proliferating cancer cells [26,27]. These results indicate that in cancer cells, which have hijacked normal cell functions to support rapid proliferation, upregulation of glutamine and glycine uptake promote proliferative activity. This, therefore, supports the idea that increasing the metabolism of these amino acids in skin wound cells could similarly lead to an increase in proliferation and ATP production.

The role of nutritional glycine supplementation on angiogenesis has also been closely studied recently, yielding an unclear picture of its effects. The totality of the studies suggests that low levels of glycine supplementation promote angiogenesis, while high levels inhibit it [28]. Herein, we posit that the variability in results obtained across experiments could stem from differences in cell types used, culture methods, and dosing [28]. Another report found that high levels of glycine inhibited angiogenesis and granulation tissue deposition in wound healing [29]. Yet another study found that glycine did not affect angiogenesis in ovarian or endometrial grafts but did inhibit apoptosis [30]. Collectively, these results suggest that the beneficial effect of glycine predicted by the methods in this thesis might depend on the dosage used. It may require balancing the effects on angiogenesis with the beneficial impacts on proliferation in other cell types and the inhibition of apoptosis. There is also a previous study conducted in 1980 that found that peritoneally injected glycine in mice showed improvements in wound healing [31].

Glutamine was predicted to have a positive effect on wound healing through its impact on metabolism, but this was not confirmed in this study, which focused on keratinocytes. Importantly, however, wound healing involves more than just keratinocytes, and the current literature indicates that the effect of glutamine on other cell types, such as fibroblasts, macrophages, and vascular endothelial cells, might be positive [32,33,34]. Similarly, in vivo studies have found that glutamine supplementation can positively affect wound healing [8]. The modeled effects might therefore still have their anticipated impact on wound healing and the more general metabolism of wound cells.

Several limitations of the mathematical model used must also be addressed to facilitate future improvements. First, a steady state was assumed, which is necessary to perform metabolic flux analysis as conducted in this study. This assumption requires that all reaction rates remain constant over time and is widely used in biological systems where it is expected that changes occur slowly compared to the dynamics of metabolism. This condition may not be satisfied over extended periods due to increasing cell numbers, but the results can still provide guidance on which aspects of the metabolic network can be adjusted. Another limitation is the model’s assumption that wound cells form a single, homogeneous compartment. This assumption is also widely used in the literature; although different compartments (such as cytosolic and mitochondrial) could be included, additional data would be necessary to clarify new fluxes in the model. Additionally, the model used is generalized to all cells in the wound. A more tailored model specific to each cell type might yield more nuanced results, but collecting data from individual cell types in the wound is currently not feasible.

## 5. Conclusions

The process outlined by Quek et al. was used to produce a metabolic model of the wound [14]. This model was employed to run simulations using biomass and ATP synthesis as indicators for cell proliferation and migration, respectively. Sensitivity analysis identified both reactions as suitable for manipulation, as they were among those with the highest average flux. Furthermore, the simulations revealed that increasing glycine and glutamine uptake fluxes could serve as potentially beneficial interventions for chronic skin wound healing, both under normoxic and hypoxic conditions closely associated with these wounds. Additional exploration of the model suggested that enhancing cofactors related to coenzyme A could also lead to improvements in the two markers relevant to chronic skin wound healing. Simulations conducted with the model indicated that proliferation and ATP production are closely linked, suggesting that increasing one typically increases the other.

Further research could involve examining results across different cell lines associated with skin wounds, such as dermal fibroblasts and vascular endothelial cells. Additionally, the model, which specifically encompasses the set of metabolic reactions alongside their constraints and stoichiometric coefficients, could be more precisely tailored to keratinocytes and other cell types related to chronic skin wounds. In vivo studies could also be conducted using identified amino acids to assess improvements in actual chronic skin wounds. Specifically, investigations to determine the most effective route of administration (intravenous, intraperitoneal, and topical) could be performed. A dosage study could also be conducted to establish the optimal dose for enhancing chronic skin wounds. It is hoped that the outcomes of this study will enable the swift implementation and improvement of the skin wound healing process in patients.

## Data Availability

Data are available from the corresponding author upon reasonable request.

## Supplementary Materials

The following supporting information can be downloaded at: https://www.mdpi.com/article/10.3390/biology14060722/s1, Figure S1: Calculated fluxes for each reaction in the order they appear in Table S3. Key reaction categories are identified on the graph; Figure S2: Average and standard deviation in flux for the first eleven sets of reactions; Figure S3: Average and standard deviation in flux for the remaining sets of reactions except transport; Figure S4: Average and standard deviation in flux for extracellular metabolite transport reactions, the transport reactions are graphed separately due to the large number of reactions and their importance in the construction of the model being explored; Figure S5: Summary of the 10 reactions with the highest average flux, with their standard deviations provided as error bars; Figure S6: Summary of the 10 reactions with the highest standard deviations normalized to average flux; Figure S7: Results for low oxygen conditions. Percent difference in flux through a group of representative cellular reactions; Figure S8: Results for low-oxygen conditions. Percentage difference in flux through a group of representative cellular reactions; Table S1: Measured metabolites; Table S2: Values from the Quek paper that were used as the average inputs for the measured metabolites; Table S3: Summary of reactions grouped by function; Table S4: Comparison of calculated fluxes to the fluxes calculated in the Quek paper. Table S5: Total flux for measured extracellular metabolites. A positive number indicates flux into the cell, while a negative number indicates flux out of the cell; Table S6: Average biomass in the simulations with the highest biomass production, average biomass in the 20 simulations with the highest ATP production, and average biomass in all 1098 simulations; Table S7: Total flux for measured extracellular metabolites. A positive number indicates flux into the cell, while a negative number indicates flux out of the cell; Table S8: Total flux for measured extracellular metabolites. A positive number indicates flux into the cell, while a negative number indicates flux out of the cell; Table S9: Total flux for measured extracellular metabolites associated with maximizing biomass. A positive number indicates flux into the cell, while a negative number indicates flux out of the cell; Table S10: 10 reactions with the highest percent difference in the 20 simulations with the highest ATP production versus the average for those reactions in all of the simulations; Table S11: Ten reactions with the highest percentage difference in the 20 simulations with the highest biomass production vs. the average for those reactions in all of the simulations.
