# Supplementary material for: Optimizing Cellular Metabolism Through Mass Balance Analysis to Improve Skin Wound Healing
Source: Biology (Basel). 2025 Jun 18;14(6):722. doi: 10.3390/biology14060722 (PMC12189431; doi:10.3390/biology14060722)
Supplement: Supplementary file 1 [file biology-14-00722-s001.zip › biology-3643189-supplementary.pdf]

Supplementary Material

Supplement A: Comparison of metabolic flux results with those presented in the Quek paper.

**Measured metabolites**
All 20 amino acids except cysteine, and in addition to the regular amino acids the glutamax (L-alanyl-L-glutamine) dipeptide
Biomass
Lactate
Glucose
Oxygen
Ammonia

Table S1: Measured metabolites.

**Measured metabolite**	**Flux from Quek paper (μmol/g of dry weight(gDW)/hr)**
Alanine	-213.3
Arginine	20.5
Asparagine	2.6
Aspartate	9.3
Glucose	623.3
Glutamine	-398.9
Glutamate	-6.4
Glycine	-7.1
Histidine	3.5
Isoleucine	4.8
Lactate	-1257
Leucine	7.5
Lysine	11.9

Methionine	5.5
Ammonia	-213.2
Oxygen	569.8
Phenylalanine	2.6
Proline	-2.4
Serine	37.4
Threonine	9.4
Tryptophan	1.7
Tyrosine	4.7
Valine	13.2
Glutamax	449.9
Biomass	0.0213

Table S2: Values from the Quek paper that were used as the average inputs for the measured metabolites

In this step, flux values were obtained for all 357 reactions in the system. The calculated fluxes from using the measured fluxes (the fluxes reported by Quek et al.). according to the order in **Table S3** are presented in **Figure S3**. Of the reactions presented in **Figure 1**, the flux through oxidative phosphorylation was the highest followed by those in glycolysis. In total, the Quek paper lists the results of metabolic flux analysis for 12 key reactions. The set of reactions in the Quek paper was used to check the agreement of this paper's model with that of the Quek Paper's model. Of those 12 reactions, 10 of the calculated fluxes were within the range listed in the Quek paper. Those results that were not within the ranges provided by the Quek paper were only outside the range by a very small amount. These results are presented in **Table S4**.

**Reaction Type**	**Reaction number**
Biomass	50
Glycolysis	96, 134, 147, 175, 196, 260, 262, 263, 265, 289, 344
Citric Acid Cycle	21, 32, 69, 141, 185, 207, 256, 335, 336
Oxidative Phosphorylation	48, 71, 72, 97, 98
Pentose Phosphate Pathway	145, 165, 227, 264, 324, 325, 337, 341, 342
Glycogenesis	146
Glycogenolysis	266
Gluconeogenesis	258
Cholesterol Synthesis	80, 86, 87, 89, 181, 182, 191, 200, 215, 273, 333, 334

Steroid Synthesis	2, 51, 54, 92, 201, 300, 301, 307
Fatty Acid Synthesis	16, 17, 52, 93, 94, 129, 130, 131, 132, 133
Fatty Acid Catabolism	12, 18, 95, 173, 174, 219, 220, 277
Phospholipid Metabolism	8, 27, 40, 41, 56, 57, 58, 59, 60, 64, 88, 143, 167, 217, 218, 259, 267, 268, 274, 275, 276, 284, 285, 288, 326, 331, 332
Amino Acid Synthesis	28, 42, 74, 75, 81, 152, 157, 180, 213, 226, 261, 270, 285, 287, 295, 296, 327, 329, 340
Amino Acid Catabolism	4, 5, 6, 7, 11, 35, 36, 45, 46, 47, 84, 136, 139, 140, 149, 154, 155, 156, 160, 166, 176, 177, 178, 187, 192, 194, 198, 204, 206, 216, 221, 222, 223, 224, 225, 243, 244, 245, 251, 252, 270, 284, 291, 292, 293, 294, 297, 298, 330, 346
Urea Cycle	38, 144
Purine Synthesis	24, 25, 26, 30, 31, 158, 189, 283, 299
Purine Catabolism	135
Pyrimidine Synthesis	43, 55, 82, 83
Transport	1, 6, 29, 33, 37, 44, 49, 68, 77, 78, 79, 91, 99, 100, 101, 102, 103, 104, 105, 106, 107, 108, 109, 110, 111, 112, 113, 114, 115, 116, 117, 118,119, 120, 121, 122, 123, 124, 125, 126, 127, 128, 137, 138, 142, 151, 153, 159, 161, 162, 163, 170, 171, 172, 179, 183, 184, 186, 188, 195, 197, 199, 202, 203, 205, 214, 228, 229, 230, 239, 240, 241, 242, 253, 257, 269, 271, 272, 281, 282, 286, 290, 302, 303, 304, 305, 308, 309, 310, 311, 312, 313, 314, 315, 316, 317, 318, 328, 339, 347, 348, 352, 353, 355
Nucleoside Phosphate Maintenance	22, 23, 70, 76, 85, 90, 150, 164, 190, 231, 232, 233, 234, 235, 236, 237, 238, 250, 275, 320, 321, 322, 323, 343, 345, 350
Carboxylic Acid Dissociation	169

Table S3: Summary of reactions grouped by function

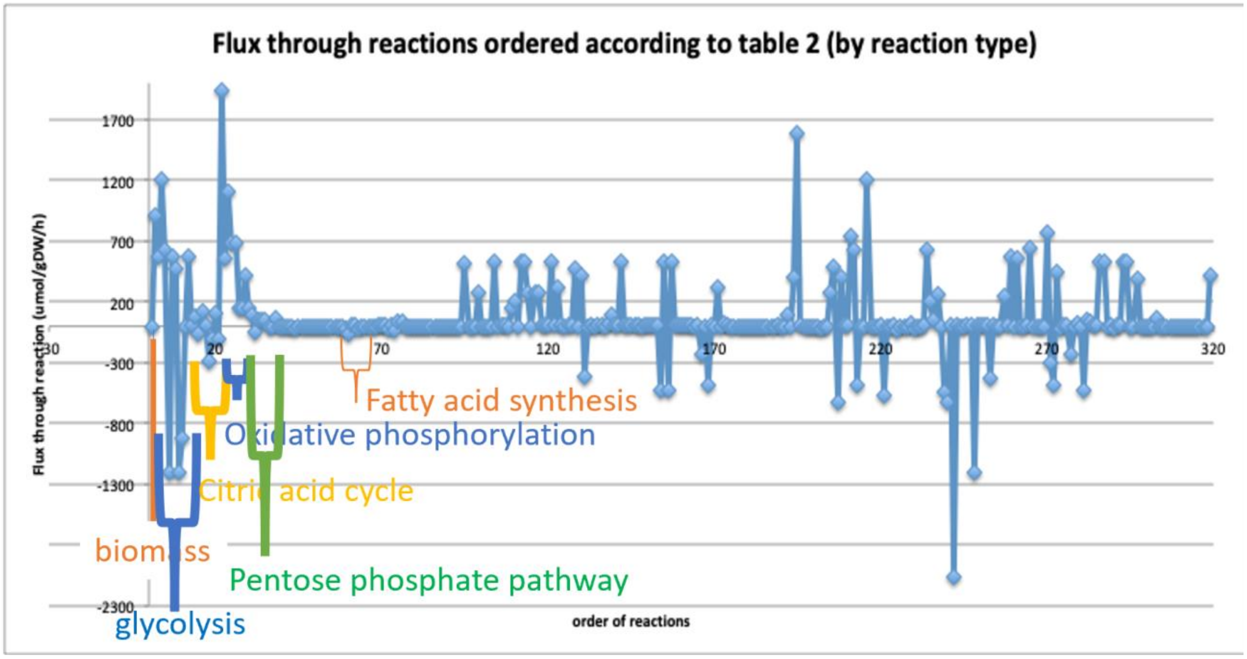

Figure S1: Calculated fluxes for each reaction in the order they appear in Table S3. Key reaction categories are identified on the graph.

Reactions	Calculated Average (μmol/gDW/h)	Standard Deviation (μmol/gDW/h)	Quek paper 95% confidence range (μmol/gDW/h)
Hexokinase	623.3	11.9	599-645
Pyruvate Kinase	986.6	33.0	787-2623
Pyruvate Dehydrogenase	18.2	53.9	33-241
Lactate Dehydrogenase	-1257	24.2	-1257- -1163
G6P Dehydrogenase	219.2	29.5	0-605
Isocitrate Dehydrogenase	3.6	51.5	0-204
2-Oxoglutarate Dehydrogenase	69.7	19.6	0-211
Succinate Dehydrogenase	102.5	19.7	10-222
ATP Synthase	1899.1	124.3	1822-4014
ATP Maintenance	347.1	28.3	0-3560
Alanine Transport (In)	236.6	58.5	161-240
Glutamine Transport (in)	51	54.5	-15-50

Table S4: Comparison of calculated fluxes to the fluxes calculated in the Quek paper.

Supplement B: Convergence of model when only extracellular metabolite inputs are varied.

The results in this section are important in order to select a number of simulations per run which gives consistent results from one run to the next. Therefore, the number of simulations per run needs to be selected so that the standard deviation among different runs is below some threshold value, which for this paper is chosen as 5%. This section presents the effect on biomass when 24 of the measured metabolites (inputs to the model) are all varied at once. The result of the simulations is shown in **Figure S2** below. From **Figure S2**, one can see that convergence is good at about 1000 simulations per run and quantitatively the standard deviation was less than 5% of the average. Convergence here means that biomass production predicted from the various runs became closer to each other as the number of simulations per run is increased. Due to the sufficient convergence observed, 1098 simulations per run was chosen for subsequent studies.

Supplement C: Sensitivity analysis.

The first thing explored when all of the input extracellular metabolites were varied for each simulation, was the effect on calculated flux variability for all 357 reactions in the system. These results help identify which inputs have the most impact on metabolic fluxes and thus are most desirable for manipulation. This data is summarized in **Figures S4-8**. For **Figures S4 and S5** the reactions were broken down into 11 categories in each of the graphs, and **Figure S6** contains the results for all of the exchange reactions. **Figure S7** contains the largest average fluxes out of the 357 reactions. Notably, biomass and ATP synthase were among the reactions with the highest average flux, corresponding to two of the markers for proliferation and cell migration respectively. These markers were chosen because of their key role in the wound-healing process. **Figure S8**, shows the 10 highest standard deviations normalized by average flux. Of note is the presence of several reactions involved in fatty acid metabolism. The first two reactions [reactions number 13 and 182] which tower above the rest in standard deviation are both involved in the production of ketone bodies, while one of them is also involved in cholesterol synthesis.

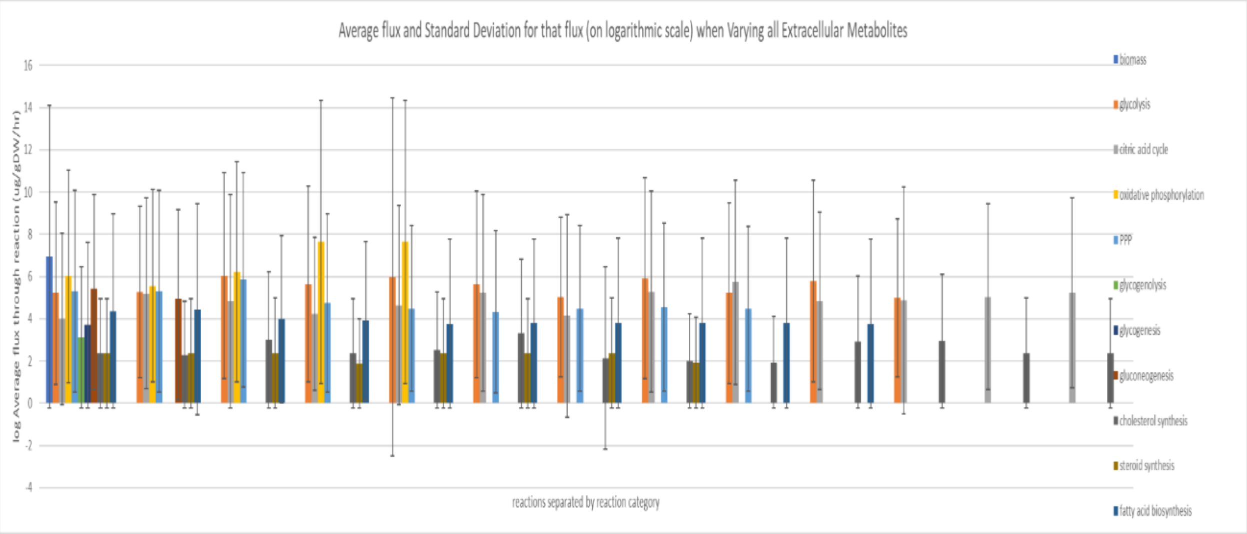

Figure S2: Average and standard deviation in flux for the first eleven sets of reactions

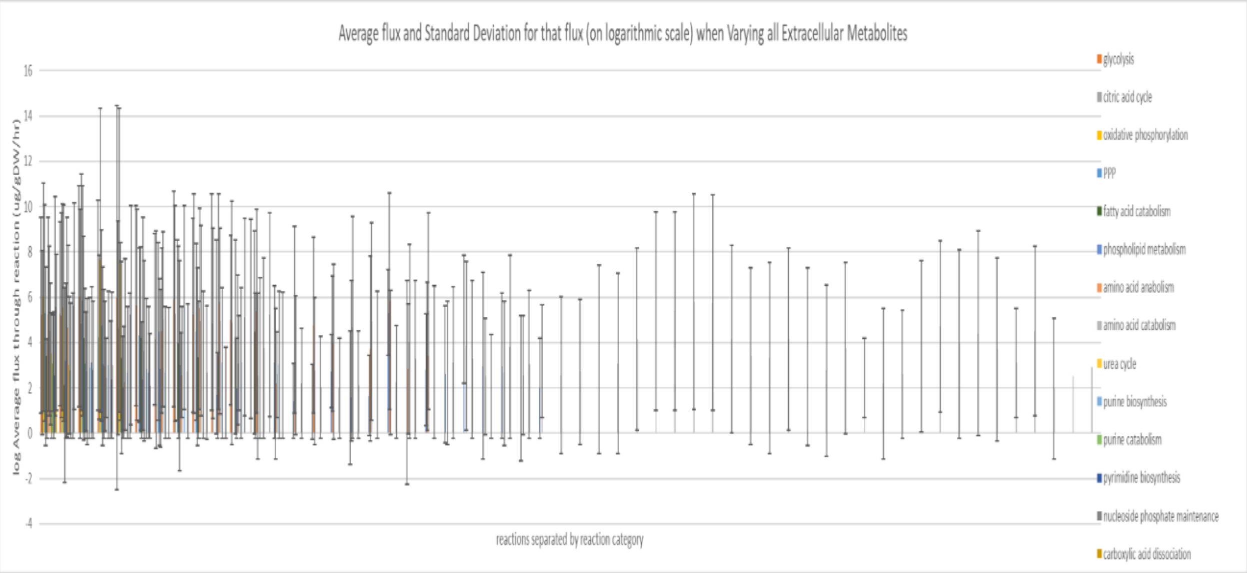

Figure S3: Average and standard deviation in flux for the remaining sets of reactions except transport

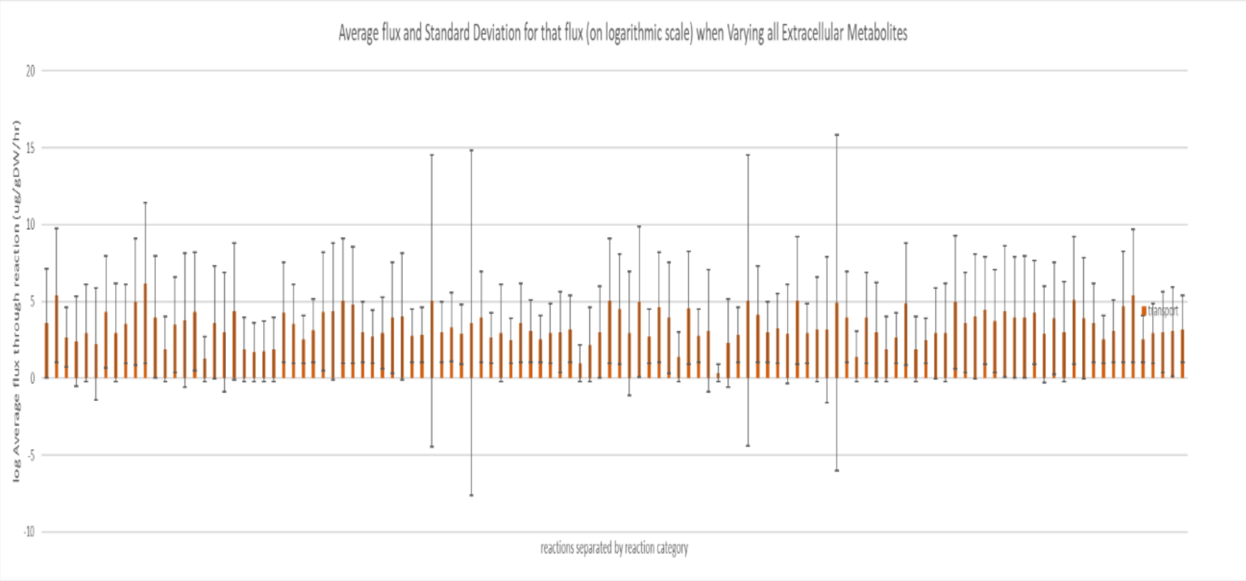

Figure S4: Average and standard deviation in flux for extracellular metabolite transport reactions, the transport reactions are graphed separately due to the large number of reactions and their importance in the construction of the model being explored

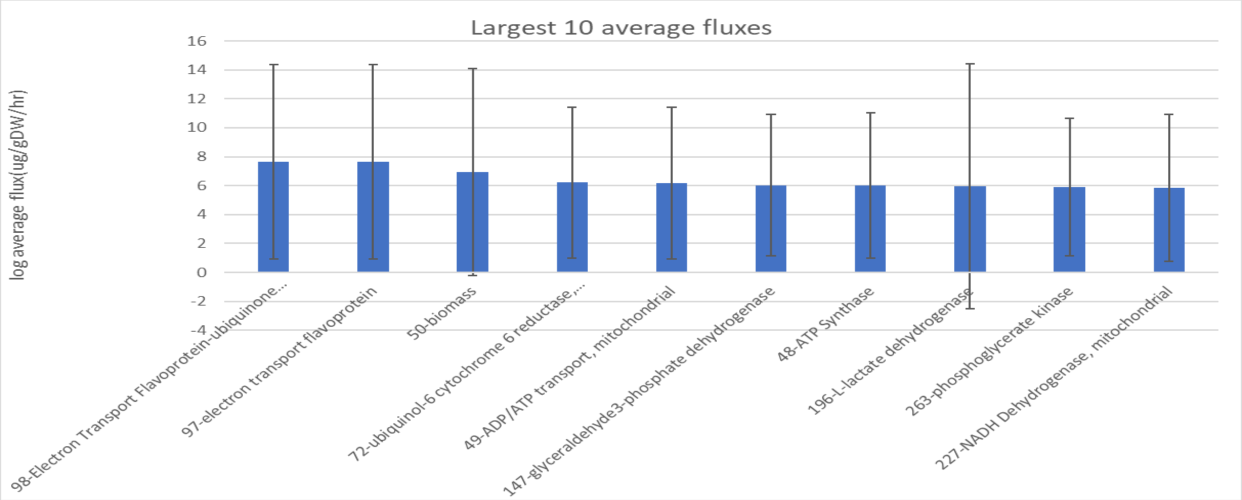

Figure S5: Summary of the 10 reactions with the highest average flux, with their standard deviations provided as error bars.

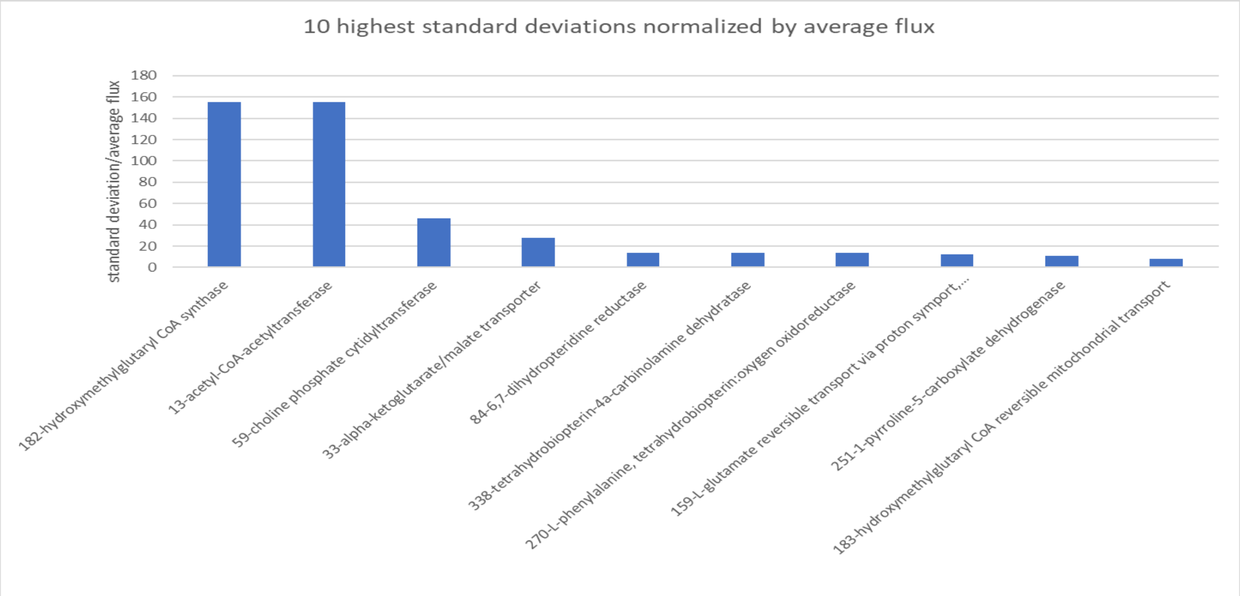

Figure S6: Summary of the 10 reactions with the highest standard deviations normalized to average flux

Supplement D: Additional figures and tables for the effect of varying all extracellular metabolites on ATP and biomass production.

Extracellular metabolite uptake rate	Average for 20 simulations with highest ATP (μmol/gDW/hr)	Average for all 1098 simulations (μmol/gDW/hr)
Alanine uptake	236.70	237.83
Arginine uptake	19.99	20.42
Asparagine uptake	2.61	2.67
Aspartate uptake	9.22	9.29
Glucose uptake	707.21	621.59
Glutamine uptake	43.72	52.56
Glutamate uptake	99.51	273.72
Glycine uptake	1.45	.52
Histidine uptake	3.64	3.50
Isoleucine uptake	4.74	4.80
Lactate Release	-1257	-1257
Leucine uptake	7.34	7.52
Lysine uptake	11.61	11.92

Methionine uptake	5.40	5.51
Ammonia Release	-213.2	-213.2
Oxygen uptake	677.61	569.24
Phenylalanine uptake	2.57	2.59
Proline Release	-2.45	-2.39
Serine uptake	38.88	37.41
Threonine uptake	9.71	9.31
Tryptophan uptake	1.73	1.70
Tyrosine uptake	4.89	4.70
Valine uptake	13.07	13.24
Glutamax uptake	444.13	450.48

Table S5: Total flux for measured extracellular metabolites. A positive number indicates flux into the cell, while a negative number indicates flux out of the cell.

	Average biomass for the subset of 20 simulations with the highest biomass production (μmol/gDW/hr)	Average biomass for the 20 simulations with the highest ATP production (μmol/gDW/hr)	Average biomass for all 1098 simulations (μmol/gDW/hr)
Average biomass	56.25	31.79	11.42

Table S6: Average biomass in the simulations with the highest biomass production, average biomass in the 20 simulations with the highest ATP production, and average biomass in all 1098 simulations

Extracellular metabolite uptake rate	Average flux for the 20 simulations with the highest biomass production (μmol/gDW/h)	Average flux for the 20 simulations with the highest ATP production (μmol/gDW/h)	Average for all 1098 simulations (μmol/gDW/h)
Alanine uptake	230.27	236.70	237.83
Arginine uptake	19.90	19.99	20.42
Asparagine uptake	2.59	2.61	2.67
Aspartate uptake	9.42	9.22	9.29
Glucose uptake	766.27	707.21	621.59
Glutamine uptake	37.71	43.72	52.56
Glutamate Release	-237.94	99.51	273.72

Glycine uptake	2.57	1.45	.52
Histidine uptake	3.45	3.64	3.50
Isoleucine uptake	4.71	4.74	4.80
Lactate Release	-1257	-1257	-1257
Leucine uptake	7.65	7.34	7.52
Lysine uptake	12.02	11.61	11.92
Methionine uptake	5.53	5.40	5.51
Ammonia Release	-213.2	-213.2	-213.2
Oxygen uptake	545.08	677.61	569.24
Phenylalanine uptake	2.64	2.57	2.59
Proline Release	-2.38	-2.45	-2.39
Serine uptake	38.18	38.88	37.41
Threonine uptake	9.44	9.71	9.31
Tryptophan uptake	1.66	1.73	1.70
Tyrosine uptake	4.79	4.89	4.70
Valine uptake	13.05	13.07	13.24
Glutamax uptake	438.54	444.13	450.48

Table S7: Total flux for measured extracellular metabolites. A positive number indicates flux into the cell, while a negative number indicates flux out of the cell.

Supplement E: Maximizing ATP and biomass under restricted uptake conditions.

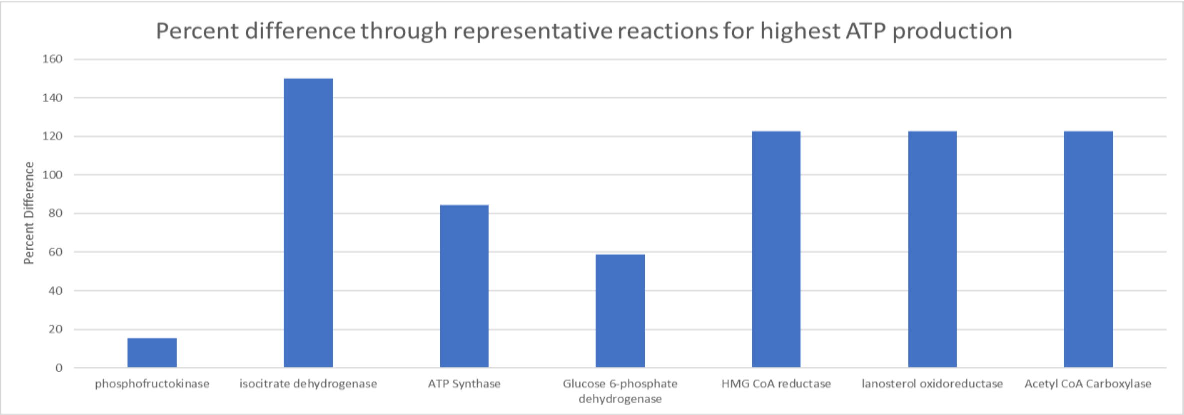

Figure S7: Results for low oxygen conditions. Percent difference in flux through a group of representative cellular reactions.

Extracellular metabolite uptake rate	Total flux (μmol/gDW/h)	Average for all 1098 reactions (μmol/gDW/h)
Alanine uptake	254.58	237.34
Arginine uptake	20.81	20.47
Asparagine uptake	2.61	2.58
Aspartate release	-9.12	-9.29
Glucose uptake	774.59	622.87
Glutamine uptake	54.55	53.70
Glutamate release	-761.74	-264.10
Glycine uptake	3.80	1.71
Histidine uptake	3.53	3.50
Isoleucine uptake	4.96	4.80
Lactate release	-1257	-1257
Leucine uptake	7.42	7.54
Lysine uptake	11.97	11.91
Methionine uptake	5.44	5.52
Ammonia uptake	213.2	213.2
Oxygen uptake	56.98	56.98
Phenylalanine uptake	2.56	2.60
Proline release	-2.49	-2.40
Serine uptake	36.33	37.48
Threonine uptake	9.19	9.36
Tryptophan uptake	1.72	1.70
Tyrosine uptake	4.61	4.70

Valine uptake	13.40	13.17
Glutamax uptake	456.23	450.34

Table S8: Total flux for measured extracellular metabolites. A positive number indicates flux into the cell, while a negative number indicates flux out of the cell.

Extracellular metabolite uptake rate	Average total flux for the 20 simulations with the highest biomass production (µmol/gDW/h)	Total flux (µmol/gDW/h)	Average for all 1098 reactions (µmol/gDW/h)
Alanine uptake	255.1388	254.58	237.34
Arginine uptake	20.83421	20.81	20.47
Asparagine uptake	2.61257	2.61	2.58
Aspartate release	-9.10503	-9.12	-9.29
Glucose uptake	774.7078	774.59	622.87
Glutamine uptake	56.57182	54.55	53.70
Glutamate release	-760.628	-761.74	-264.10
Glycine uptake	3.805244	3.80	1.71
Histidine uptake	3.512339	3.53	3.50
Isoleucine uptake	4.941114	4.96	4.80
Lactate release	-1257	-1257	-1257
Leucine uptake	7.553081	7.42	7.54
Lysine uptake	11.94609	11.97	11.91
Methionine uptake	5.432794	5.44	5.52
Ammonia uptake	213.2	213.2	213.2
Oxygen uptake	56.98	56.98	56.98
Phenylalanine uptake	2.607053	2.56	2.60
Proline release	-2.46746	-2.49	-2.40
Serine uptake	36.26926	36.33	37.48
Threonine uptake	9.159428	9.19	9.36
Tryptophan uptake	1.729166	1.72	1.70

Tyrosine uptake	4.622324	4.61	4.70
Valine uptake	13.46088	13.40	13.17
Glutamax uptake	455.655	456.23	450.34

Table S9: Total flux for measured extracellular metabolites associated with maximizing biomass. A positive number indicates flux into the cell, while a negative number indicates flux out of the cell.

Reactions	Percent difference
3-hydroxyisobutyryl-CoA hydrolase	-323959.7
Acyl-CoA dehydrogenase (isobutyryl-CoA) mitochondrial	-323959.7
3-hydroxyisobutyrate-dehydrogenase	-323959.7
3-hydroxyacyl-CoA dehydratase (3-hydroxyisobutyryl CoA) (mitochodrial)	-323959.7
2-oxoisovalerate dehydrogenase	-323959.7
Methylmalonate-semialdehyde dehydrogenase	-323959.7
Valine reversible mitochondrial transport	-323959.7
Valine transaminase, mitochondrial	-323959.7
Cystathione beta-synthase	-3729.62
Cystathione g-lyase	-3729.62

Table S10: 10 reactions with the highest percent difference in the 20 simulations with the highest ATP production versus the average for those reactions in all of the simulations.

Reactions	Percent difference
3-hydroxyisobutyryl-CoA hydrolase	-323150.9
Acyl-CoA dehydrogenase (isobutyryl-CoA) mitochondrial	-323150.9
3-hydroxyisobutyrate-dehydrogenase	-323150.9
3-hydroxyacyl-CoA dehydratase (3-hydroxyisobutyryl CoA) (mitochodrial)	-323150.9
2-oxoisovalerate dehydrogenase	-323150.9
Methylmalonate-semialdehyde dehydrogenase	-323150.9
Valine reversible mitochondrial transport	-323150.9
Valine transaminase, mitochondrial	-323150.9

Cystathione beta-synthase	-3740.023
Cystathione g-lyase	-3740.023

Table S11: Ten reactions with the highest percent difference in the 20 simulations with the highest biomass production versus the average for those reactions in all of the simulations.

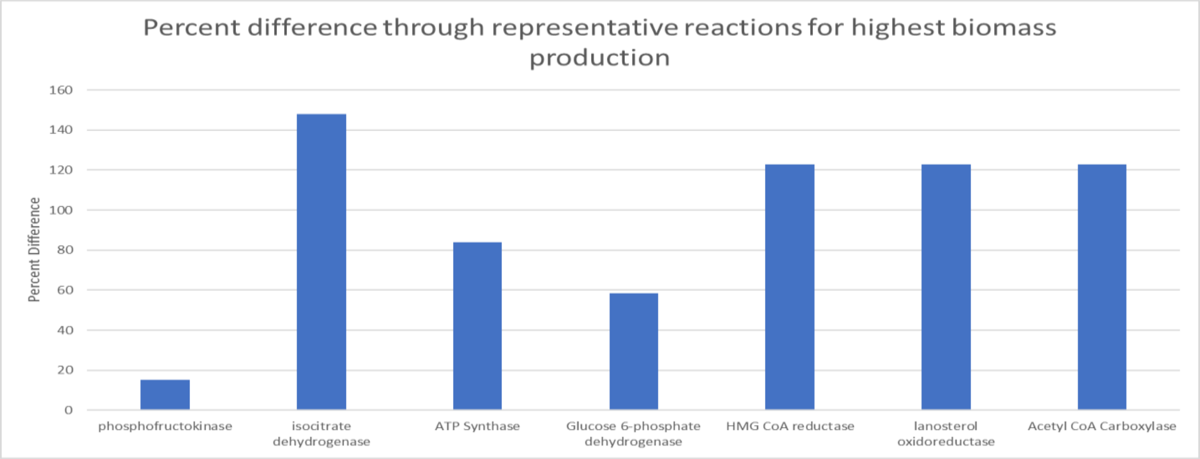

Figure S8: Results for low oxygen conditions. Percent difference in flux through a group of representative cellular reactions.
